# Cinnamon Polyphenol Extract Inhibits Hyperlipidemia and Inflammation by Modulation of Transcription Factors in High-Fat Diet-Fed Rats

**DOI:** 10.1155/2017/1583098

**Published:** 2017-03-15

**Authors:** Zeynep Tuzcu, Cemal Orhan, Nurhan Sahin, Vijaya Juturu, Kazim Sahin

**Affiliations:** ^1^Division of Biology, Faculty of Science, Firat University, Elazig, Turkey; ^2^Department of Animal Nutrition, Faculty of Veterinary Science, Firat University, Elazig, Turkey; ^3^Research and Development, OmniActive Health Technologies Inc., Morristown, NJ, USA

## Abstract

We evaluated the effects of cinnamon polyphenol extract on hepatic transcription factors expressions including SREBP-1c and LXR-*α* in rats fed high fat diet (HFD). Twenty-eight Wistar rats were allocated into four groups: (i) normal control: animals fed with normal chow; (ii) cinnamon: animals supplemented with cinnamon polyphenol; (iii) HFD: animals fed a high-fat diet; and (iv) HFD + cinnamon: animals fed a high-fat diet and treated with cinnamon polyphenol. Obesity was linked to hyperglycemia, hyperlipidemia, and oxidative stress as imitated by elevated serum glucose, lipid profile, and serum and liver malondialdehyde (MDA) concentrations. Cinnamon polyphenol decreased body weight, visceral fat, liver weight and serum glucose and insulin concentrations, liver antioxidant enzymes, and lipid profile (*P* < 0.05) and reduced serum and liver MDA concentration compared to HFD rats (*P* < 0.05). Cinnamon polyphenol also suppressed the hepatic SREBP-1c, LXR-*α*, ACLY, FAS, and NF-*κ*B p65 expressions and enhanced the PPAR-*α*, IRS-1, Nrf2, and HO-1 expressions in the HFD rat livers (*P* < 0.05). In conclusion, cinnamon polyphenol reduces the hyperlipidemia, inflammation, and oxidative stress through activating transcription factors and antioxidative defense signaling pathway in HFD rat liver.

## 1. Introduction

Obesity is an important health problem that characterized excessive fat accumulation in the body resulting from an imbalance in energy intake and expenditure [1]. It is known to be a risk factor for numerous metabolic complaints such as diabetes, atherosclerosis, hyperlipidemia, and cancer [2, 3]. Consumption of high-fat diet causes a leading obesity and obesity-related complications including hyperlipidemia and oxidative stress [4, 5]. Liver fat synthesis is an extremely modified metabolic pathway which is vital for actual low-density lipoprotein production and is, therefore, important for energy delivery to other tissues [6]. Transcription factors such as the sterol regulatory element-binding protein 1 (SREBP-1) and liver X receptors (LXRs) and several enzymes including ATP-citrate lyase (ACL), acetyl-CoA carboxylase (ACC), and fatty acid synthase (FAS) have vital roles in this process [7–9]. SREBP-1 may control the ectopic accumulation of fat and may set the target gene FAS, an important enzyme that controls the amount of fatty acid synthesis [10, 11]. Obesity-induced insulin resistance triggers inflammation in the liver through the accumulation of reactive oxygen species that trigger nuclear factor kappa beta (NF-*κ*B) pathway [12]. Besides systemic and hepatic fat metabolism deterioration, inflammation is a major factor underlying liver damage in diabetes [13, 14]. The peroxisome proliferator-activated receptor *α* (PPAR*α*), which is extremely expressed in the liver, shows a vital role in the modulation of liver lipid metabolism [15].

Some potent drugs carry the risk of side effects on the central nervous system and the cardiovascular system in the treatment of obesity [16, 17]. Natural products can show an obvious role in the prevention of obesity and associated metabolic diseases. Cinnamon has been used as spice for a long time [18]. In addition it is gained from the inner bark of tropical evergreen cinnamon plants [18]. There are two main types of cinnamon: True or Ceylon cinnamon* (Cinnamomum verum and C. zeylanicum)* and cassia cinnamon (*C. aromaticum* and* C. burmannii*). That cinnamon as a dietary supplement has antioxidant, anti-inflammation, and presently discovered antihyperlipidemia and antiobesity effect properties [19, 20]. Experimental and clinical studies have shown that cinnamon could be attributed to its beneficial effects on hyperlipidemia and glucose utilization [21, 22]. A study reported that cinnamon increases insulin sensitivity and liver glycogen by modulating insulin signaling and glycogen synthesis in insulin-resistant rats [22]. Research in animal models has also proven that cinnamon effectively prevents obesity caused by high-fat diets [23]. However, the underlying exact mechanisms are still unclear. Hence, in this study, the effects of cinnamon polyphenol extract on PPAR*α*-mediated target genes involved in glucose and lipid metabolism, including SREBP-1c, LXRs, ATP-citrate lyase (ACLY), and FAS, and expression of molecular targets of inflammation (NF-*κ*B) and antioxidant status (Nrf2 pathway) in the liver were examined to investigate more detailed mechanisms in the improvement of fatty liver with high-fat diet.

## 2. Materials and Methods

### 2.1. Animals

Twenty-eight Wistar rats (weighing 180 ± 20 g) were kept under a 12 h light/dark cycle at 22 ± 2°C, with feed and water ad libitum. Rats had free access to diet and water. Rats received humanitarian care according to standards defined in the “Guidelines for the Care and Use of Laboratory Animals” delivered by the National Academy of Sciences and published by the National Institutes of Health and permitted by the Ethical Commission of the Firat University, Elazig, Turkey. The composition of diets (control and HFD) is shown in Table 1. For obesity induction, animals were fed with HFD for 12 weeks and compared with rats fed normal diet.

### 2.2. Experimental Diets and Design

After acclimatization for 2 weeks, 28 rats were randomly allocated into four groups, with 7 rats in each group: (i) normal control group: animals fed with normal chow (12% of calories as fat) throughout the experimental period of 12 weeks; (ii) cinnamon group: rats fed with normal chow and administered with cinnamon polyphenol (100 mg/kg b.wt.) throughout the experimental period of 12 weeks; (iii) HFD group: rats fed with high-fat diet [42% of calories as fat] throughout the experimental period of 12 weeks; and (iv) HFD + cinnamon group: rats fed a high-fat diet and administered with cinnamon polyphenol throughout the experimental period of 12 weeks. Rats were orally treated with cinnamon polyphenol extract [100 mg/kg b.wt. dissolved in 5% dimethyl sulfoxide (DMSO)] daily by oral gavage in olive oil (1 ml/kg b.wt./day) to the end of the experiment. The amount of cinnamon polyphenol extract used was based upon an earlier study presenting a positive result of 100 mg of cinnamon per kilogram on diabetic rats [24]. The control rats in this study received similar amounts of sunflower oil by gavage. Cinnamon product (Product Code: 33002; Lot Number: CINP10001b) obtained from* Cinnamomum zeylanicum* by the aqueous-alcoholic extraction used in this study was provided by OmniActive Health Technologies Ltd. (Pune, India). The test compound contains 18.41% total polyphenols and it is light to dark reddish brown free flowing powder with an astringent taste. The quality of cinnamon polyphenol extract was confirmed to comply with strict quality control measures and found free of endotoxin and heavy metals.

At the end of the study, the blood was collected by cardiac puncture after an overnight fast and all rats were sacrificed by cervical dislocation. The visceral fat and liver samples were removed and weighed after sacrificing the animals.

### 2.3. Biochemical Estimations

Serum was prepared by centrifuging the blood at 3,000 ×g for 10 minutes and used for biochemical parameters and malondialdehyde (MDA) analyses. Sera samples were prepared by centrifuging the blood at 3,000 ×g for 10 min and used for the analyses of biochemical parameters and MDA. Serum parameters were determined using an automated analyzer (Samsung LABGEOPT10, Samsung Electronics Co., Suwon, Korea). Repeatability and device/method exactness of LABGEOPT10 were documented according to the IVR-PT06 guideline. The concentration of serum insulin was measured with the Rat Insulin kits (Linco Research Inc., St. Charles, MO, USA) by ELISA (Elx-800, Bio-Tek Instruments Inc., Vermont, USA). The sensitivity of the assays for insulin was 0.36 ng/ml. The interassay and intra-assay coefficients of variation were 5.3% and 7.5% for insulin. Liver MDA levels were determined according to the method described by Karatepe [25] by HPLC with a Shimadzu UV-Vis SPD-10 AVP detector, a CTO-10 AS VP column, and a mobile phase comprised of 30 mM KH_2_PO_4_ and methanol (82.5 : 17.5, v/v, pH 3.6) at a flow rate of 1.2 ml/min. Column effluents were monitored at 250 nm. Liver homogenate (10%, w/v) was prepared in 10 mM phosphate buffer (pH 7.4) and centrifuged at 13,000 ×g for 10 minutes at 4°C. The resulting supernatant was collected and kept at −80°C for MDA estimation.

Total antioxidant capacity (TAC) was determined by dark blue-green color reduction 2,2′-azino-bis 3-ethylbenzothiazoline-6-sulfonate (ABTS) by antioxidants to its colorless form via the antioxidants in the sample [26]. In this analysis, ABTS is incubated with potassium persulfate to produce ABTS oxidation. Briefly, 10 mg of ABTS was dissolved in 10 mL of an aqueous solution containing 2.5 mmol/L potassium persulfate and allowing the mixture to stand in the dark at room temperature for one to four hours before use. For the study of samples, ABTS oxidized stock solution was diluted with deionized water to an absorbance of 0.70 at 734 nm. After addition of 1 mL diluted ABTS with 10 *μ*L of serum oxidized, the absorbance readout was taken ten minutes after the first mixing. The results were expressed in mmol Trolox E/L.

Activity of total superoxide dismutase (SOD) in the homogenized liver tissue (in 20 mM HEPES (N-2 hydroxyethyl piperazine-N′-2-ethanesulfonic acid) buffer, 1 ‎mM ethylene glycol tetraacetic acid, 210 mM mannitol, and 70 mM sucrose, pH 7.2, per g of tissue) was determined by a commercial kit (Cayman Chemical, Ann Arbor, MI, USA) according to the manufacturer's instructions. The supernatant was collected after centrifugation at 12.000*g* for 20 min at 4°C. The supernatant was purified from the salt by passing through a Sephadex G-25 column. The samples were also treated with a mixture of ethanol-chloroform (2 : 1, v/v) and distilled water to remove hemoglobin and red blood cells and the absorbance plate was read on a reader (Bio-Tek Instruments, Inc., Vermont, USA) at 450 nm. The results were expressed as units per mg protein (U/mg protein) using standard calibration curve. Catalase (CAT) activity was also determined in homogenized tissue (50 mM potassium phosphate, 1 mM EDTA, pH 7, in cold buffer, per tissue) using a commercial kit (Cayman Chemical, Ann Arbor, MI, USA) according to the manufacturer's instructions. The supernatant was collected after centrifugation at 12,000*g* for 20 minutes at 4°C. A formaldehyde solution was used as standard. The absorbance of standard and samples was taken at 540 nm using a plate reader (Bio-Tek Instruments, Inc. Vermont, USA). Catalase activity was expressed as nmol/min/mg protein using standard calibration curve. The activity of glutathione peroxidase (GSHPx) was analyzed according to the manufacturer's instructions (Cayman Chemical, Ann Arbor, MI, USA). Liver tissue was homogenized with the Polytron Homogenizer in cold buffer (50 mM Tris-HCl, pH 7.5, 5 mM EDTA, and 1 mM dithiothreitol) per tissue and then subjected to centrifugation at 10,000*g* for 15 minutes at 4°C. This method is based on the oxidation of NADPH to NADP^+^, which is accompanied by an absorbance drop at 340 nm and GSHPx activity was measured by initiating the reaction with 2.4 mM cumene hydroperoxide. One unit is defined as the amount of enzyme that oxidizes 1 *μ*mol of NADPH per min at 25°C. The absorbance was read every minute at 340 nm using a plate reader (Bio-Tek Instruments, Inc., Vermont, USA) to obtain at least 5 time points. The GSHPx activity was calculated in nmol/min/mg of protein using standard calibration curve.

### 2.4. Western Blot Analyses

Protein extraction was performed by standardizing the liver in 1 ml of ice-cold hypotonic buffer (buffer A) containing 10 mM HEPES (2-(4-(2-hydroxyethyl)-1-piperazinyl) ethane sulfonic acid, PH 7.8, 10 mM KCl, 2 mM MgCl2, 1 mM dithiothreitol (DTT), 0.1 mM EDTA, and 0.1 mM phenylmethylsulfonyl fluoride (PMSF) for Western blot analysis. The homogenate was mixed with 80 *μ*l of 10% Nonidet P-40 (NP-40) solution and then centrifuged at 14,000 ×g for 2 minutes. The precipitates were washed once with 500 *μ*L buffer A and 40 *μ*L 10% NP-40, centrifuged, and resuspended in a 200 *μ*L buffer containing 50 mM HEPES, pH 7.8, 50 mM KCl, 300 mM NaCl, 0.1 mM EDTA, 1 mM DTT, 0.1 mM PMSF, and 20% glycerol) and recentrifuged at 14,800 ×g for 5 min. The concentration of the protein was determined according to the procedure described by Lowry using a protein assay kit (Sigma, St. Louis, MO, USA). The supernatant was collected and used for the determination of SREBP-1c, LXRs, ACLY, FAS, NF-*κ*B p65, PPAR*α*, p-IRS-1, Nrf-2, and HO-1 according to the previously described method [27]. Briefly, 50 *μ*g of proteins was electrophoresed and then transferred to a nitrocellulose membrane (Schleicher and Schuell Inc., Keene, NH, USA). The phosphorylated form of antibodies against SREBP-lc, LXRs, ACLY, FAS, NF-*κ*B p65, PPAR*α*, p-IRS-1, Nrf-2, and HO-1 (Abcam, Cambridge, UK) was diluted (1 : 1000) in the same buffer containing 0.05% Tween-20. Protein loading was controlled using monoclonal mouse antibody against *β*-actin (A5316; Sigma). The bands were examined densitometrically using ImageJ, an image analysis system (National Institute of Health, Bethesda, USA).

### 2.5. Statistical Analysis

Data were stated as mean ± SE. The alteration among groups was analyzed using one-way analysis of variance (ANOVA) followed by the Tukey post hoc test (SAS Institute: SAS User's Guide: Statistics), and *P* < 0.05 was considered statistically significant.

## 3. Results

### 3.1. Effect of Cinnamon Extract on Body Weight and Visceral Fat in HFD-Fed Rats

The effect of cinnamon polyphenol extract treatment on the final body weight, feed consumption, and visceral fat and liver mass was shown in Table 2. HFD feeding increased final body weight, visceral fat, and liver weight by 33.1%, 258.3%, and 34.8% and decreased feed intake by 16.9% as compared to the control rats (*P* < 0.001). Although the cinnamon polyphenol extract treatment decreased body weight, visceral fat, and liver weight by 8.4%, 36.6%, and 17.7% in the HFD-fed rats (*P* < 0.001), the HFD-fed rats treated with cinnamon still had a final body weight and visceral fat higher than those of the control rats (*P* < 0.05). No significant difference was found in the feed intake between HFD-fed rats and HFD-fed rats treated with cinnamon polyphenol extract (*P* > 0.05).

### 3.2. Effect of Cinnamon Extract on Biochemical Parameters in HFD-Fed Rats

Table 3 shows the effect of cinnamon polyphenol extract on supplementation on carbohydrate and lipid profile in HFD-fed rats. As seen in the table, HFD feeding increased the serum levels of glucose and insulin, free fatty acid (FFA), total cholesterol, HDL-C, and LDL-C, as well as TG in HFD rats (*P* < 0.001). The hypertriglyceridemia and elevated lipid indicators in HFD-fed rats were reduced with cinnamon polyphenol extract supplementation. HFD did not cause a significant increase in aspartate transaminase (AST) and alanine transaminase (ALT) in the duration of the treatment, and the levels remained more or less unaffected in cinnamon polyphenol extract supplemented rats (*P* > 0.05).

### 3.3. Effect of Cinnamon Extract on Antioxidant Status in HFD-Fed Rats

Serum and liver MDA levels increased by 158.8% and 81.7% (*P* < 0.001; Table 4) and serum TAC, liver SOD, CAT, and GSHPx decreased by 67.6, 54.7, 34.4%, and 56.4% upon obesity induction. The cinnamon polyphenol extract treatment caused 23.3% and 25.4% reduction in serum and liver MDA concentration and elevation in serum TAC, liver SOD, CAT, and GSHPx of 91.2, 62.6%, 21.9%, and 36.0% in the HFD-fed rats (*P* < 0.001), which was like the control group (*P* > 0.05).

### 3.4. Effect of Cinnamon Extract on Protein Levels in HFD-Fed Rats

**Figure 1:**
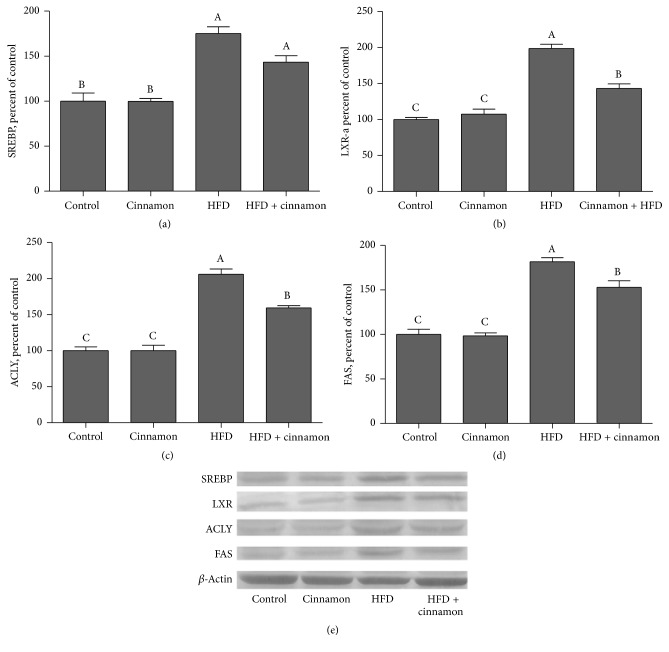
Hepatic SREBP-1c, LXRs, ACLY, and FAS expression levels in cinnamon polyphenol supplemented high-fat diet- (HFD-) fed rats and control groups. (a)–(d) show the expression level of SREBP-1c, LXRs, ACLY, and FAS in various groups. The intensity of the bands shown in (e) was quantified by densitometric analysis. Data are expressed as a ratio of normal control value (set to 100%). Each bar represents the mean and standard error of mean. Blots were repeated at least 3 times (*n* = 3) and only a representative blot is shown in (e). *β*-Actin was included to ensure equal protein loading.

SREBP-1c, LXRs, ACLY, and FAS expression in the HFD-fed rats increased by 75.1%, 98.7%, 106.0%, and 81.7% in liver (Figure 1), respectively (*P* < 0.0001 for all). SREBP-1c, LXRs, ACL, and FAS expression decreased by 18.1%, 27.9%, 22.7%, and 15.8%, respectively (*P* < 0.05 for all), when the HFD rats were treated with cinnamon polyphenol extract. All remained lower as compared to the control rats (*P* > 0.05 for both).

**Figure 2:**
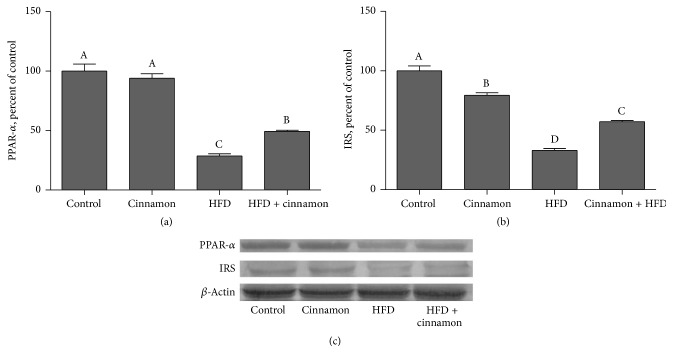
Hepatic PPAR*α* and IRS expression levels in cinnamon polyphenol supplemented high-fat diet- (HFD-) fed rats and control groups. (a) and (b) show the expression level of PPAR*α* and IRS in the groups. The intensity of the bands shown in (c) was quantified by densitometric analysis. Data are expressed as a ratio of normal control value (set to 100%). Each bar represents the mean and standard error of mean. Blots were repeated at least 3 times (*n* = 3) and only a representative blot is shown in (c). *β*-Actin was included to ensure equal protein loading.

PPAR*α* and IRS expression in liver in the HFD group were 71.3% and 67.0% lower than those in the control group (Figure 2;* P* < 0.001 for both). Despite the respective 1.72- and 1.73-fold increase in PPAR*α* (Figure 2(a)) and IRS (Figure 2(b)) expression with cinnamon polyphenol extract treatment (*P* < 0.001 for both), PPAR*α* and IRS expression levels still remained lower compared to the control group (*P* < 0.001 for both).

**Figure 3:**
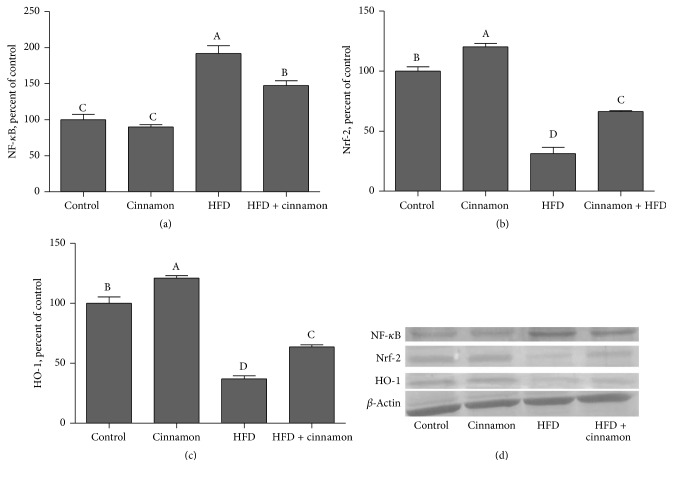
Hepatic NF-*κ*B p65, Nrf2, and HO-1 expression levels in cinnamon polyphenol supplemented high-fat diet- (HFD-) fed rats and control groups. (a)–(c) show the expression level of NF-*κ*B p65, Nrf2, and HO-1 in the groups. The intensity of the bands shown in (d) was quantified by densitometric analysis. Data are expressed as a ratio of normal control value (set to 100%). Each bar represents the mean and standard error of mean. Blots were repeated at least 3 times (*n* = 3) and only a representative blot is shown in (d). *β*-Actin was included to ensure equal protein loading.

Expression of NF-*κ*B increased by 92.2% in the liver in the HFD rats (Figure 3(a);* P* < 0.001). The cinnamon polyphenol extract treatment partially restored NF-*κ*B expression levels in liver (by 23.3%;* P* < 0.05; Figure 3(a)) as compared to the control group. The induction of obesity was associated with 68.7 and 63.0% reduction in expression of Nrf2 and HO-1 in liver (*P* < 0.001; Figures 3(b) and 3(c)), respectively. The cinnamon polyphenol extract treatment partially elevated the expression of Nrf2 and HO-1 in the liver (by 111.7% and 72.1%;* P* < 0.001; Figures 3(b) and 3(c)).

## 4. Discussion

High-fat dietary intake leads to insulin resistance (IR) and altered glucose and lipid metabolism [28]. Cinnamon polyphenols can respond to IR and are therefore useful because of their insulin-enhancing and antioxidant properties [29]. Cinnamon extracts have been recognized as in vitro and in vivo insulin sensitizers [22, 30]. The adverse effects of HFD/HFD on brain insulin signal changes were alleviated by the use of cinnamon, which suggests that cinnamon is associated with whole body insulin sensitivity and related changes, including hippocampal synaptic plasticity and cognition in the brain of neuroprotective effects [31, 32]. Consistent with previous studies, our results demonstrated that cinnamon polyphenol extract supplementation improved body weight, visceral fat, and carbohydrate metabolism including glucose, insulin, and free fatty acid and lipid profiles (TC, TG, and HDL-C) and lipid peroxidation and antioxidant enzymes in the HFD-fed rats [19, 33–36]. Qin et al. [28] reported that cinnamon polyphenol extract increased the use of insulin-regulated glucose in rats. In addition, Mang et al. [21] reported that cinnamon prevents IR by partially increasing insulin signaling pathway with high fructose diet.

Cinnamon extracts have also been shown to be useful in decreasing fasting plasma glucose, cholesterol, and triglycerides in diabetic patients [37]. Similarly, application of cinnamon extract reduced liver MDA levels in carbon tetrachloride-poisoned rats and improved SOD, CAT, and GSHPx activities [38]. Cinnamon has been shown to prevent hyperlipidemia and improved glucose tolerance in rats fed fructose/high fat [22, 39]. However, a direct association between cinnamon polyphenol intake and regulated SREBP-1c, LXRs, ACLY, and FAS expression by cinnamon polyphenol in the HFD-fed rats has yet to be established. Previous studies have shown that SREBP-1c has a regulatory role in the synthesis of lipogenic enzymes such as FAS, which inhibits TG accumulation in the liver, in fatty acid synthesis and lipid metabolism [8]. LXRs are also transcription factors that regulate fatty acid and cholesterol homeostasis and are expressed mainly in the liver and other tissues involved in lipid metabolism [40]. ACLY play a crucial role in obesity-related complications in glucose and lipid homeostasis of mice liver [9]. An animal study has shown activation of LXR protection effects in obesity induced by high-fat diet [41]. In the present study, we demonstrated for the first time that cinnamon polyphenol intake significantly reduced the expression of hepatic SREBP-1c, LXRs, ACLY, and FAS. There are no earlier studies associated with examining the effects of cinnamon polyphenol treatment on the expression of SREBP-1c, LXRs, ACL, and FAS in rats fed HFD to compare with this study. Nevertheless, it was reported that cinnamon prevented the hyperlipidemia in fructose-fed rats and improved glucose tolerance [39].

Peroxisome proliferator-activated receptors (PPARs), transcriptional factors complicated in the modulation of IR and adipogenesis, play key roles in regulating carbohydrate and lipid metabolism [42]. Activation of PPAR reduces serum triglycerides and raises serum HDL-cholesterol concentrations [43], whereas activation of PPAR*γ* increases insulin sensitivity and causes antidiabetic effects [44]. IRS-1 plays an essential role in the pathway of insulin-stimulated signal transduction and binds the insulin receptor to its ultimate biological activities by a series of intermediates [45]. In a prior report, we showed that HFD in diabetic rats decreased PPAR*γ* expression in the adipose tissue and reduced expression of IRS-1 in the liver and kidney [46]. In this study, cinnamon polyphenol increased PPAR*α* and IRS expression in the liver; this may have potential insulin sensitizing effect and may increase IR in a rat obesity model. In accordance with our findings, Sheng et al. [42] showed that the cinnamon extract could induce expression of PPAR*γ* and PPAR*α* both in vitro and in vivo in mouse adipose cells. Similarly, Qin et al. [47] found that cinnamon extract supplementation resulted in reduced expression of interleukin-1*β* (IL-1*β*), IL-6, and tumor necrosis factor-*α* (TNF-*α*) mRNA while increasing expression of IR, IRS1, and IRS2 in hamster enterocytes.

NF-*κ*B is a transcription factor that is responsible for controlling a DNA transcription and comprises cellular responses to various stimuli including free radicals. Kuhad and Chopra [48] reported that the signal transduction pathway for the activation of transcription factor NF-*κ*B was evoked by reactive oxygen species associated with hyperglycemia and by advanced glycosylated end products [48]. In the situation of oxidative stress and numerous cytokines, NF-*κ*B is quickly released from I*κ*B in order to stimulate the expression of chemotactic and matrix proteins of various cytokines involved in inflammation, immunological responses, and/or proliferation [49]. In the present study, cinnamon polyphenol reduced liver of NF-*κ*B expression in rats fed HFD (Figure 3). In a previous study, we have reported that HFD consumption enhances inflammation and NF-*κ*B activation [50]. Fan et al. [51] showed that activity of NF-*κ*B increased in rats fed HFD. But there was no earlier study studying the effects of cinnamon polyphenol on the NF-*κ*B p65 in the liver with which to compare this study. Nevertheless, in a previous study, it was shown that cinnamon-based treatment induced inhibition of NF-*κ*B and neuroinflammation and supported our present findings [52].

Another important mechanism contributing to cinnamon antiobesity is the upregulation of antioxidant-dependent proteins. We found that expression of the proteins Nrf2 and HO-1 increased in HFD rats with cinnamon intake, indicating that this antioxidant mechanism may underlie reduced levels of lipid peroxidation in liver tissues. Nrf2 transcription factor is one of the most important antioxidant defense mechanisms that protect cells and tissues from various oxidative stresses [53]. Specifically, Nrf2 induces the expression of genes encoding antioxidant proteins, including HO-1, by binding to the antioxidant response element [54]. HO-1 is reported to be a highly effective therapeutic target for protection against oxidative stress and damage. HO-1 also is one of phase II detoxifying enzymes and exerts a strong antioxidant effect, and it is regulated by the redox-sensitive transcription factors. In addition, Nrf2 likely interferes with lipogenic and cholesterolemic pathways, inhibiting lipid accumulation and oxidative stress in the mouse liver after administration of HFD [55]. In the current study, cinnamon polyphenol d increased Nrf2 and HO-1 expression in liver of rats fed by HFD (Figure 3). Tuzcu et al. [50] showed similar reductions in Nrf2 and HO-1 expressions as increased serum MDA in HFD-fed rats. In addition, cinnamaldehyde, an important flavor component in cinnamon essential oil upregulated Nrf2 expression, stimulated its translocation to the nucleus, and increased HO-1, NQO1, CAT, and GPx1 expression under high glucose conditions [7]. Wondrak et al. [56] reported that cinnamaldehyde and cinnamon extract upregulated cellular protein levels of Nrf2 in human colon cancer cells and recognized Nrf2 targets involved in the antioxidant response including HO-1 and gamma-glutamyl-cysteine synthetase.

## 5. Conclusions

In conclusion, cinnamon polyphenol has been reported to have several beneficial effects on obesity through the modulation of transcription factors including SREBP-1c, LXRs, NF-*κ*B, and Nrf2 and several enzymes such as ACLY and FAS and insulin resistance, glucose, and lipid metabolism and antioxidant status. Cinnamon polyphenol may have a potential use in the management of hyperglycemia and hyperlipidemia.

## Figures and Tables

**Table 1:** Composition of diets (g/kg diet) fed to rats.

	Control	HFD
Casein	200.0	200.0
Starch	579.5	150.0
Sucrose	50.0	149.5
Soybean oil	70.0	—
Beef tallow	—	400.0
Cellulose	50.0	50.0
Vitamin-mineral premix^*∗*^	45.0	45.0
l-Cysteine	3.0	3.0
Choline bitartrate	2.5	2.5

^*∗*^The vitamin-mineral premix provides the following (per kg): all-*trans*-retinyl acetate, 1.8 mg; cholecalciferol, 0.025 mg; all-*rac*-a-tocopherol acetate, 12.5 mg; menadione (menadione sodium bisulfate), 1.1 mg; riboflavin, 4.4 mg; thiamine (thiamine mononitrate), 1.1 mg; vitamin B-6, 2.2 mg; niacin, 35 mg; Ca-pantothenate, 10 mg; vitamin B-12, 0.02 mg; folic acid, 0.55 mg; *d*-biotin, 0.1 mg; manganese (from manganese oxide), 40 mg; iron (from iron sulfate), 12.5 mg; zinc (from zinc oxide), 25 mg; copper (from copper sulfate), 3.5 mg; iodine (from potassium iodide), 0.3 mg; selenium (from sodium selenite), 0.15 mg; choline chloride, 175 mg.

**Table 2:** Effect of cinnamon polyphenol extract supplementation on the body weight, visceral fat, and the liver weight in rats fed with HFD for 12 weeks.

Item	Groups			
	Control	Cinnamon	HFD	HFD + cinnamon
Final BW (g)	301.43 ± 5.27^C^	298.86 ± 6.50^C^	401.29 ± 5.07^A^	367.71 ± 2.75^B^
Feed intake (g/d)	22.77 ± 0.44^A^	22.96 ± 0.40^A^	18.93 ± 0.44^B^	19.91 ± 0.41^B^
Visceral fat (g)	6.62 ± 0.45^C^	6.35 ± 0.33^C^	23.72 ± 1.58^A^	15.04 ± 0.48^B^
Liver (g)	11.63 ± 0.25^C^	11.79 ± 0.42^C^	15.68 ± 0.38^A^	12.90 ± 0.38^B^

HFD, high-fat diet; data are expressed as mean ± SEM of 7 rats from each group. A, B, and C: means in the same row with different superscripts are significant (*P* < 0.05).

**Table 3:** Effects of cinnamon polyphenol extract biochemical parameters levels in rats fed with HFD for 12 weeks.

Item	Groups			
	Control	Cinnamon	HFD	HFD + cinnamon
Glucose (mg/dl)	75.86 ± 2.62^C^	76.57 ± 3.34^C^	200.86 ± 3.97^A^	158.43 ± 2.07^B^
Insulin (ng/mL)	1.61 ± 0.04^C^	1.55 ± 0.04^C^	8.21 ± 0.29^A^	4.47 ± 0.23^B^
FFA (mM)	1.74 ± 0.11^C^	1.48 ± 0.06^C^	5.03 ± 0.14^A^	2.25 ± 0.07^B^
T-C (mg/ml)	66.51 ± 5.58^B^	53.26 ± 1.90^B^	91.71 ± 2.28^A^	61.43 ± 1.81^B^
HDL-C (mg/dl)	15.29 ± 0.57^BC^	13.57 ± 0.77^C^	22.57 ± 0.53^A^	18.57 ± 0.43^AB^
TG (mg/dl)	25.86 ± 1.26^C^	24.14 ± 1.81^C^	57.57 ± 2.08^A^	41.85 ± 1.49^B^
AST (U/L)	146.413 ± 4.40	142.71 ± 4.30	157.00 ± 8.28	154.22 ± 5.38
ALT (U/L)	83.65 ± 6.59	81.86 ± 4.18	89.14 ± 4.39	86.43 ± 5.16

HFD, high-fat diet; FFA, free fatty acids; T-C, total cholesterol; HDL-C, high-density lipoprotein cholesterol; TG, triglycerides; AST, aspartate aminotransferase; ALT, alanine transferase. Data are expressed as mean ± SEM of 7 rats from each group. A, B, and C: means in the same row with different superscripts are significant (*P* < 0.05).

**Table 4:** Effects of cinnamon polyphenol extract on oxidative stress and antioxidant status rats fed with HFD for 12 weeks.

Item	Groups			
	Control	Cinnamon	HFD	HFD + cinnamon
Serum MDA (nmol/mL)	0.68 ± 0.04^C^	0.64 ± 0.03^C^	1.76 ± 0.03^A^	1.35 ± 0.03^B^
Liver MDA (nmol/mg protein)	1.97 ± 0.06^C^	1.90 ± 0.13^C^	3.58 ± 0.09^A^	2.67 ± 0.04^B^
Serum TAC (nmol Trolox Equiv. per mg protein)	1.76 ± 0.06^A^	1.88 ± 0.12^A^	0.57 ± 0.07^C^	1.09 ± 0.10^B^
Liver SOD (U/mg protein)	202.29 ± 5.80^A^	206.14 ± 7.31^A^	91.71 ± 3.98^C^	149.14 ± 2.03^B^
Liver CAT (U/mg protein)	349.86 ± 11.97^A^	353.85 ± 14.06^A^	229.57 ± 4.30^C^	279.82 ± 9.62^B^
Liver GSHPx (U/mg protein)	53.67 ± 4.25^A^	54.86 ± 3.56^A^	23.42 ± 2.56^C^	31.86 ± 2.48^B^

HFD, high-fat diet; MDA, malondialdehyde; TAC, total antioxidant capacity; SOD, superoxide dismutase; CAT, catalase; GSHPx, glutathione peroxidase. Data are expressed as mean ± SE of 7 rats from each group. A, B, and C: means in the same row with different superscripts are significant (*P* < 0.05).
